# Perventricular device closure of the ventricular septal defects in children-first experience in the United Arab Emirates

**DOI:** 10.1186/s13019-024-02959-6

**Published:** 2024-07-10

**Authors:** Aleksandr Omelchenko, Neerod Kumar Jha, Nishant Shah, Antoine AbdelMassih, Juan Pablo Montiel, Benedict Raj Rajkumar

**Affiliations:** 1 Division of Pediatric Cardiac Surgery, Institute of Cardiac Sciences, Sheikh Khalifa Medical City, Abu Dhabi, 768010, United Arab Emirates; 2 Division of Pediatric Cardiology, Institute of Cardiac Sciences, Sheikh Khalifa Medical City, Abu Dhabi, United Arab Emirates

**Keywords:** Perventricular, Ventricular septal defect, Heart, Children, Device

## Abstract

**Background:**

Ventricular septal defect (VSD) is the most common congenital cardiac malformation, accounting for approximately 30% of congenital heart defects. Conventional surgical repair using cardiopulmonary bypass is invasive and associated with morbidities and prolonged hospital stay. With the advent of interventional approaches and availability of different occluding devices, the technique of perventricular device closure is evolving and being implemented successfully in larger groups of patients. We present herein, our initial experience of perventricular device closure for the ventricular septal defects in children to assess risks and benefits.

**Methods:**

From March, 2023 to February, 2024, we have performed perventricular closure of ventricular septal defects in 13 children, under guidance of transesophageal echocardiography without cardiopulmonary bypass support. The median age at operation was 2 year (range 1.3–10 years) with the median body weight 11 kg (range 8.7–16.6 kg). Sixty-nine percent were males. The ventricular septal defect sizes ranged from 2.7 to 6 mm (mean 4.7 mm). Seven defects were perimembranous, four sub-aortic and two were muscular. One patient also underwent pulmonary artery de-banding with pulmonary artery balloon angioplasty and other one patent ductus arteriosus ligation, concomitantly. For defect closure, we used ventricular septal defect occlusion device (MemoPart™, Lepu Medical Technology Company, China) through a 3-cm skin incision in the lower- third of the sternum. The device sizes ranged from 5 to 8 mm (mean 6.9+-1.8 mm) and all patients except for two required symmetrical devices.

**Results:**

All patients underwent device closure successfully. The procedural duration ranged between 32 and 52 min. None of the patients required cardiopulmonary bypass. The mean ventilation time and intensive care unit stay was 3 and 24 h, respectively. None of the patients required inotropic support or blood transfusions. Moreover, no patients developed any arrhythmias including heart block. The average length of hospital stay was 4.4 days. At the latest follow up, there were no residual shunts, conduction disturbances, device dislodgement or major aortic or tricuspid valve complications seen in any patients. There was no mortality.

**Conclusions:**

Perventricular device closure of ventricular septal defects is a less invasive, extremely safe and effective method in children. It is associated with very fast recovery, shorter hospitalization time and better cosmetic incision. Moreover, it avoids cardiopulmonary bypass. The modifications and refinements in the design, material and implantation techniques will help in expanding the indications and prevent complications in the long-term.

## Background

Ventricular septal defect (VSD), in which 70% of the cases are perimembranous (PM) is the most common congenital cardiac malformation, accounting for approximately 30% of congenital heart defects [1]. The surgical repair through median sternotomy using cardiopulmonary bypass has been mainstay of the treatment until recently [2]. With the advent of new interventional approaches and availability of different occluding devices, the percutaneous catheter-based closure and perventricular device closure (PDC) has been developed and described in larger groups of patients [1, 3]. Herein, we present our initial experience of PDC in children, the first ever report from the ‘Middle-East’ region. The institutional review board’s approval was obtained before the study.

## Methods

### Type of study and patient selection

The retrospective chart review was done. The detailed preoperative transthoracic and pre-operative transesophageal echocardiography (TEE) evaluations including Doppler study confirmed the location and size of the VSDs, in addition to presence of associated cardiac anomalies. Indications for intervention included presence of left ventricular volume load and aortic valve prolapse with or without mild aortic regurgitation. Patients with evidence of pulmonary vascular disease, inlet extension of the PM VSD or any associated cardiac lesions such as mitral cleft or severe aortic regurgitation were excluded from the PDC. Demographic data was collected.

### Technique of PDC

**Fig. 1:**
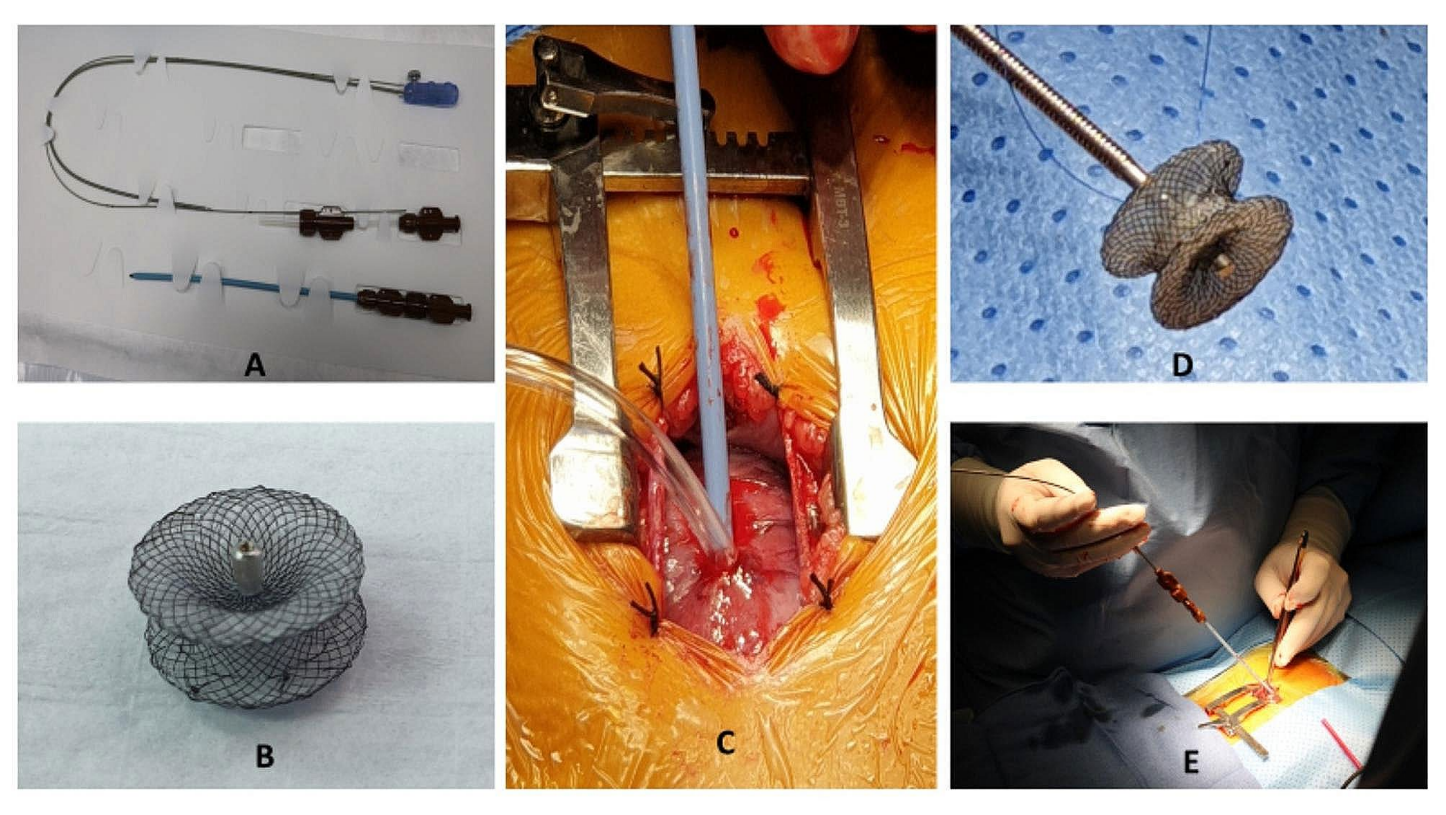
Device and the delivery system: **A**- Device introduction assembly, **B**- Symmetrical occluder device, **C**- Surgical Incision, **D**- Device mounted on the introducer, E- Insertion technique

**Fig. 2:**
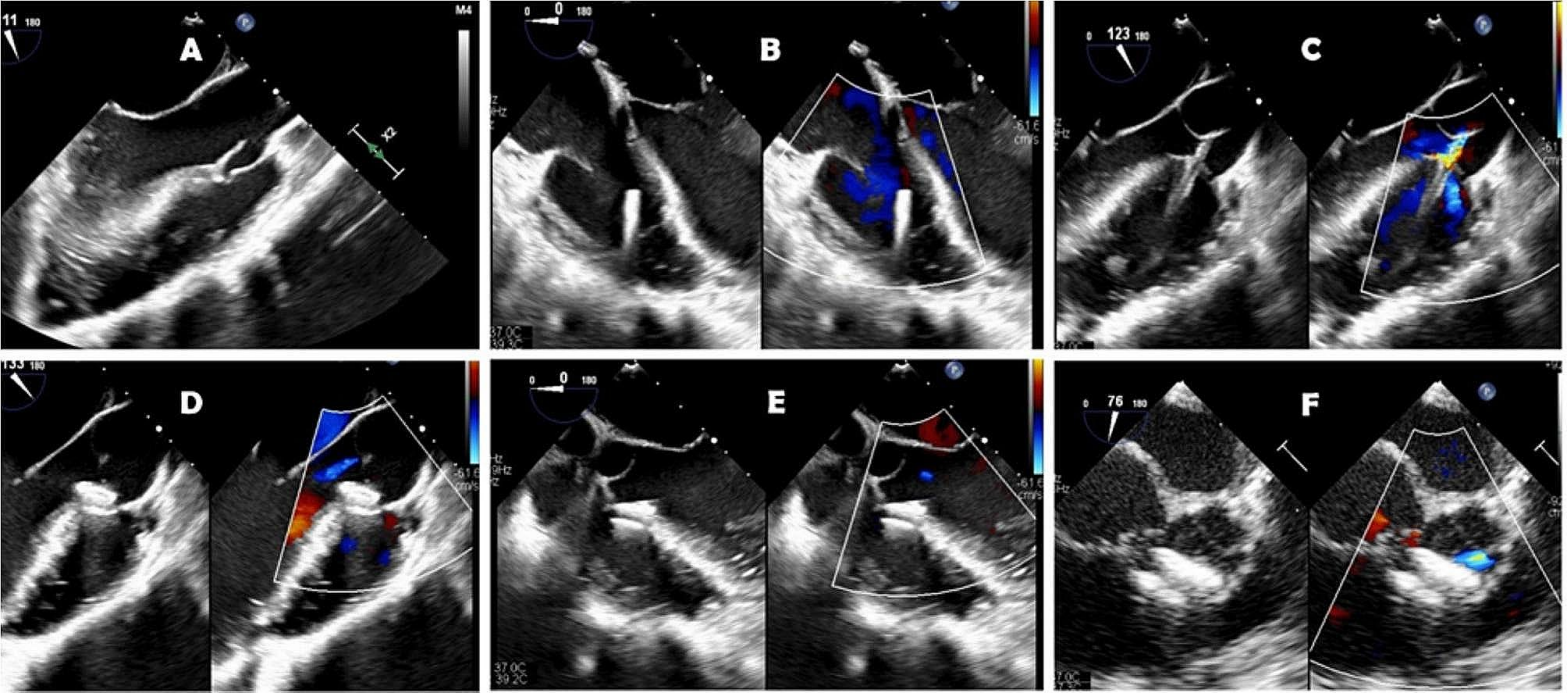
Serial transesophageal echocardiography images obtained during device implantation: **A**- prolapse of aortic valve in one patient, **B**- guide wire in the right ventricle, **C**- catheter in the left ventricular outflow tract, **D**- VSD device in the sagittal TEE view (12–130), **E**- device in five chamber view TEE angle 0, **F**- VSD device in short axis view

The patients were prepared for regular cardiac procedure under general anesthesia with backup of cardiopulmonary bypass facility. During the procedure, a small 3 cm long incision was given at the lower third of the sternum including xiphoid and a mini lower median sternotomy was performed. Then, pericardium was opened and suspended to expose the right ventricle. At this time, systemic heparin (1 mg/kg) was administered. To determine the puncture site, the free right ventricular (RV) wall was palpated lightly to locate the area of maximal thrill, corresponding to the VSD location. A purse-string suture was placed using 5 − 0 ‘Prolene’ on the RV free wall at the point of maximal thrill. A guidewire was inserted through a needle into the right ventricle perpendicularly and guided through the VSD under TEE imaging and then the delivery sheath was introduced through the dilator in to the defect. A second sheath (loader) with the VSD occlusion device mounted on the tip (MemoPart™, Lepu Medical Technology Company, China) was screwed into it and pulled back until both disks were completely open (Fig. 1). The left-side disc was deployed in the left ventricle side first and the device then was pulled back towards the septum, and right disc was deployed. The device chosen for closure was ‘oversized’ by 2 mm. In the majority of the cases (n-11), we used a symmetrical device. However, in 2 patients with a subaortic defect, an asymmetrical device was implanted. The shape and symmetry of the device to be used was decided as per location of VSD and its proximity to the aortic valve. If the aortic cusp and the VSD-margin distance was less than 2 mm, an asymmetrical device was used. The exact positioning of the device was based on the location and the direction of the prominent metallic tip which is integral part of the device. This tip guides us to keep it towards left ventricular outflow tract and also helps repositioning during rotation for suitable placement, if required. The position of the device, residual shunt, and aortic and tricuspid insufficiency were checked by the TEE during the procedure (Fig. 2). After securing the hemostasis on the right ventricular puncture site, the chest wall was closed in a routine manner. Oral aspirin was administered for 3-months in all patients. In one patient, who earlier had pulmonary artery (PA) banding, the de-banding was done and subsequently the PA was dilated at the band site using a balloon catheter introduced through the same guide wire used for device implantation. In other patient, moderate patent ductus arteriosus (PDA) was ligated during the same procedure. Both patients required limited extension of the sternotomy for better exposure.

### Statistical analysis

Data were analyzed using a commercial trial version of ‘Medcalc’. Numerical data was expressed as mean ± SD, if normally distributed, and as median and interquartile range, if not. Categorical data were expressed as number and percentage for each category. Data were presented as summary or descriptive statistics without any subgroups or intergroup differences and comparison.

## Results

A total of 13-patients underwent perventricular septal defect device closures during the study period. Indications for VSD closure were as follows: volume overload evident by mild or more left ventricular dilatation in 6 patients, aortic valve prolapse with mild aortic regurgitation (AR) in three patients, aortic valve prolapse without aortic regurgitation in one patient, mild aortic regurgitation without prolapse in one patient, large VSD and PA band in one patient and moderate PDA with VSD leading to volume overload along with failure to thrive in one patient. All patients underwent VSD closure successfully. The ventricular septal defect sizes ranged from 2.7 to 6 mm. (mean 4.7 mm). The device sizes ranged from 5 to 8 mm (mean 6.9+-1.8 mm). The mean procedural duration ranged (skin-to-skin) between 32 and 52 min. None of the patients required cardiopulmonary bypass. The mean ventilation time and intensive care unit stay was 3 and 24 h, respectively. Eight patients were extubated immediately after the procedure in the operation room. None of the patients required inotropic support or blood transfusions. Immediate post procedure TEE revealed no residual VSD shunt. Only two patients had trivial AR. However, the AR was not found in any patients during the last follow-ups. Additionally, none of the patients developed arrhythmias including heart block or right bundle branch block. There was no pericardial effusion. The mean length of hospital stay was 4.4 days. At follow up, (range 3 months-9 months) there were no residual shunts, conduction disturbances, device dislodgement or major aortic or tricuspid valve complications in all the patients. There was no mortality (Table 1).

**Table 1:** Demographic data and results

Variable		*N* = 13
Demographics		
Age (years) Median (IQR)		2 (1.3–10)
Sex N (%)	Male	9 (69)
	Female	4 (31)
Weight (Kg) Median (IQR)		11 (8.7–16.6)
VSD characteristics by TEE		
VSD type N (%)	Subaortic	4 (31)
	PM	7(54)
	Muscular	2 (15)
VSD size (mm) mean ± SD		4.7 ± 1.4
Evidence of coronary cusp prolapse with AR by TEE N (%) Only AR	Yes	3 (23)
	Yes	01 (7.6)
Evidence of Coronary cusp prolapse without AR by TEE (N %) VSD with PA band VSD with PDA	Yes	01 (7.6)
	Yes Yes	01(7.6)) 01(7.6)
Procedure details		
Aristotle complexity score mean ± SD		5.8 ± 0.3
Procedure duration (min) Median (IQR)		39 (32–52)
Device size (mm) mean ± SD		6.9 ± 1.8
Device symmetry N (%)	Symmetric	11 (85)
	asymmetric	2 (15)
Need for CPB N (%)	Yes	0 (0)
	No	13 (100)
Residual shunt (VSD or device)		
Residual shunt immediately postoperative N (%) and at follow up	Yes	0 (0)
	No	13 (100))
Hospital stay and complications		
Duration of ICU stay (days) mean ± SD		2 ± 0.8
Duration of Hospital stay (days)mean ± SD		4.4 ± 1.1
Heart block N (%)	Yes	0(0)
	No	13(100)
Mortality N (%)	Yes	0(0)
	No	13 (100)
Follow up range	3–9 months	

Abbreviations: AR: aortic regurgitation, CPB: cardiopulmonary bypass, IQR: interquartile range, m: month, N: number, SD: standard deviation, TEE: transesophageal echocardiography, VSD: ventricular septal defect, PDA: patent ductus arteriosus, PA: pulmonary artery.

## Discussion

So far, the surgical repair of the VSD on cardiopulmonary bypass was considered as the gold standard in children [2]. However, it is invasive and associated with surgical trauma, morbidity, and mortality [2–5]. The use of cardiopulmonary bypass to maintain perfusion during open heart surgery is a non-physiological state with possible severe hemodilution, acute inflammatory response and macro or micro-thrombosis which may lead to multiple organ dysfunctions (2–3, 5, 6, 7, 8). In addition, in small children, it can lead to excessive perioperative blood loss and requires a large volume of transfusion [3, 4, 8] Moreover, surgical repair of the VSD has risk of arrhythmias including heart block. The conduction system can easily be compromised with surgical stitches or wall tension [3–8].

Many studies have therefore described clinical outcomes and economic benefits of percutaneous transcatheter closure by comparing it with conventional surgical repair in selected patients [3, 8]. Although transcatheter occlusion is minimally invasive and effective, it has several undesirable aspects related to the vascular access, limited manipulation, and exposure to radiation. In addition, percutaneous catheter-based interventions in children may be difficult and carries a high risk of complications because of the large size of the sheath relative to the size of the patient body weight, size and location of the defects [3, 8].

Hence, the PDC had been introduced in a baby by Amin ‘et al’ after initial animal experiments [5]. Recently, many reports are available with the use of PDC techniques with good mid-term results for the VSDs [3–11]. Intraoperative TEE should look for the size and location of the VSD. In addition, function of the aortic valve, function and attachment of the tricuspid valve need to be assessed. As per literature, even with PDC, the risks of device dislocation, valvular regurgitation, and heart block still exists [11]. In order to avoid complications, we should look for contraindications such as VSD > 12 mm, severe pulmonary hypertension, significant aortic or tricuspid valve pathology, and endocarditis [11].

A meta-analysis of PDC for VSD closure by Hong ‘et al’ (2019) in children has described results of 15 selected studies describing 1368 patients [11]. Only 109 patients developed some complications related to the procedure and 88% complications were at intra operative stage (residual shunt 29.36%, newly AR 28.44%, AV block 9.17%, failure to establish tract 7.33% and newly TR 6.42%). Postoperative complications (11%) included newly TR 0.92%, AV Block 4.59%, occluder dislodgement 1.83%, second operation 3.67%). During follow up period the complications rates were AV block 2.75%, AR 4.59% and residual shunt 0.92% [11]. Upon occurrence of complications, removal of device and conversion to surgical method usually resolves the issue in most of the cases. However, heart block may persist.

In suitable patients, the PDC not only simplifies VSD closure but also eliminates the potential complications of cardiac catheterization and fluoroscopy because it is performed under echocardiographic guidance only. Some of contraindications of the PDC include smaller patients (less than 2.4 kg or one-month age), severe cardiac comorbidity, left ventricular outflow tract pathologies, and perimeter of prolapsing aortic cusp by more than 30%.

The device-related complications described in the literature are related to the wide range of motion of the inserted device due to weak and friable wall of the membranous septum or presence of aneurysm. Sometimes the device size may mismatch or presence of redundant tissue leads to crowding in the place making device seating inappropriate [3–10]. The relationship between the VSD and the tricuspid valve morphology is crucial and therefore a careful preoperative planning and intraoperative monitoring with TEE is important. Any impingement on the tricuspid valve, abnormal chordal attachments and aneurysmal tissues are some aspects to be considered and excluded. In our earlier experience with different patient population in other centers, we could rotate the device accordingly to position it in such patients during PDC. Therefore, in our opinion, the tricuspid redundant tissue is not a contraindication any more [9, 10]. Similarly, the aneurysmal tissue of the membranous septum is a risk factor and so far, such patients were considered unsuitable for PDC. We believe that usually the aneurysmal tissue is dense on the right side of the VSD. Therefore, if we use newer devices with larger discs on the left side of the VSD, it can occlude it completely. Currently, efforts to modify shape, curvature and materials of the devices are contributing to successful management of patients with such morphologies. An example is Cocoon device or device with larger or convex shape left -sided disc.

The occluder device can cause initial inflammatory response and subsequent fibrosis in or around the conduction system in addition to possible flattening of the device in due course [11]. Both changes may be profound if a larger device is selected. Therefore, in our opinion, the device should not be oversized by more than 2 mm for respective VSD sizes.

The PDC has been used for infant population and during combined procedure for ligation of the PDA earlier [10]. In present study, we have ligated a PDA by extending the sternal incision a bit more in one and also successfully de-banded pulmonary artery and performed subsequent angioplasty in another patient as a first step during device closure. We believe that associated lesions as described above are not contraindications to the PDC, anymore. Additionally, our findings support the concept that mild AR or prolapse is not a contraindication to the PDC, rather AR improved during follow up.

## Conclusions

Perventricular device closure of ventricular septal defects is a safe and effective method in children. It is less-invasive approach that not only avoids cardiopulmonary bypass but also provides a shorter period of rehabilitation and excellent cosmetic result.

Although the technique of perventricular device closure is safe in the mid-term follow-up, it remains unclear whether in long-term it will be without any late complication such as arrhythmia or changes in the heart function. Therefore, further experience and long-term follow-up is mandatory to comparatively evaluate the safety and applicability of this procedure. The modifications and refinements in the design, material and implantation techniques will not only help in expanding the indications of PDC but also will include more patients with septal or tricuspid aneurysmal tissues, aortic regurgitation or prolapse and other cardiac comorbidities.

## Data Availability

No datasets were generated or analysed during the current study.

## Abbreviations

VSD: ventricular septal defect
PM: perimembranous
PDC: perventricular device closure
TEE: transesophageal echocardiography
RV: right ventricle
PDA: patent ductus arteriosus
PA: pulmonary artery
AR: aortic regurgitation
